# Collisions between CO, CO$_2$, H$_2$O and Ar ice nanoparticles compared by molecular dynamics simulation

**DOI:** 10.1038/s41598-022-18039-5

**Published:** 2022-08-16

**Authors:** Maureen L. Nietiadi, Yudi Rosandi, Eduardo M. Bringa, Herbert M. Urbassek

**Affiliations:** 1 Physics Department and Research Center OPTIMAS, University Kaiserslautern, Erwin-Schrödinger-Straße, 67663 Kaiserslautern, Germany; 2 Department of Geophysics, Universitas Padjadjaran, Jatinangor, Sumedang 45363 Indonesia; 3 CONICET and Facultad de Ingenería, Universidad de Mendoza, 5500 Mendoza, Argentina; 4 Centro de Nanotecnología Aplicada, Facultad de Ciencias, Universidad Mayor, 8580745 Santiago, Chile

**Keywords:** Space physics, Materials science

## Abstract

Molecular dynamics simulations are used to study collisions between amorphous ice nanoparticles consisting of CO, CO$_2$, Ar and H$_2$O. The collisions are always sticking for the nanoparticle size (radius of 20 nm) considered. At higher collision velocities, the merged clusters show strong plastic deformation and material mixing in the collision zone. Collision-induced heating influences the collision outcome. Partial melting of the merged cluster in the collision zone contributes to energy dissipation and deformation. Considerable differences exist—even at comparable collision conditions—between the ices studied here. The number of ejecta emitted during the collision follows the trend in triple-point temperatures and increases exponentially with the NP temperature.

## Introduction

Collisions between dust particles are ubiquitous in space. Examples are provided by protoplanetary dust disks^1,2^, interplanetary dust particles in evolved planetary systems originating from comets^3,4^ or asteroid collisions^5–7^, planetary rings^8^ and even planetary nebulae^9^. In these contexts, the outcome of a collision between dust particles is of interest as it determines the evolution of the size distribution of dust particles in the population^10^; in the context of protoplanetary disks, such collisions govern the early stages of planet formation^11^.

The composition of these dust particles differs depending on the type of protostar and on the distance to it^12^. The sequence of ices is governed by the so-called ‘ice lines’; that is the distance to the star beyond which a particular ice species will condense from the gas phase and form ice particles or mantle other, more refractory, particles. The position of the ice lines is influenced by the temperature-dependent sublimation pressures of the ices, the radial pressure distribution in the protoplanetary disk, the outward diffusion of gas and the inward drift of condensed ice grains. Among the most important species are silica and water, but also carbon-containing species such as in particular CO and CO$_2$ are relevant^12^. For the example of the protosolar system, it is estimated that the water ice line is at 2 AU followed by CO$_2$ at around 10 AU^13,14^. The position of the CO ice line is further outside, at around 30 AU^15–17^.

Collisions between dust particles have mostly been studied for silica particles, both with experiments^18–20^ and simulations^21,22^. But also collisions between water ice particles were investigated^23–26^ and it was found that water ice is considerably more sticky than silica^24,27^. In atomistic simulations, it was shown that it is in particular the larger plastic deformability of water ice and its ability to melt under the collision that give rise to the large differences in the collisional behavior of silica and water ice^28^. While even nanosized silica particles may bounce from each other at sufficiently large collision velocities, ice particles will stick for all velocities apart form high-energy collisions which fragment the colliding grains.

Collisions between dust nanoparticles (NPs) consisting of other ice species—such as CO and CO$_2$—have been studied less frequently. Musiolik et al.^14^ performed collisions between 100 μm-sized CO$_2$ ice particles and found that the bouncing velocity was unexpectedly low. They observed that the collision behavior is not similar to water ice but resembles in its poor sticking characteristics more that of silica particles. Arakawa and Krijt^29^ review available data on collisions between CO$_2$ particles and discuss them in a macroscopic continuum framework^30^. They find that H$_2$O ice particles undergo high viscoelastic energy dissipation under the collision which accounts for their large bouncing velocity, while the viscoelastic dissipation of CO$_2$ is small. Kimura et al.^31^ argue that the high stickiness of water ice is caused by a melt film of molecular thickness—the so-called quasi-liquid layer^32^ or premelting^33^—that surrounds ice particles even at extremely low temperatures.

Other authors proposed that the vanishing dipole moment of CO$_2$ might be the source of the small tendency of sticking of CO$_2$ ices^34,35^. This idea was later criticized^29^ since according to macroscopic theories of bouncing^30,36^, the bouncing velocity is mainly influenced by the surface energy, but this quantity is of similar magnitude for H$_2$O and CO$_2$ ices.

In the present paper, we address the question of how collisions between ice nanoparticles consisting of CO, CO$_2$ and water differ from each other from an atomistic point of view. For comparison, we also compare to collisions of a purely van-der-Waals bonded Ar particle. In addition, since collision-induced melting is known to exert a major influence on the collision outcome, the influence of the initial NP temperature on the collision dynamics is investigated.

## Method

The four ices studied here differ strongly in their properties. Some of the relevant characteristics are assembled in Table 1: the temperature and number density at the triple point, $T_t$ and $n_t$, and at the critical point, $T_c$ and $n_c$. In addition, the ‘boiling temperature’, $T_b$, i.e. the vaporization temperature at 0.1 MPa, is listed.

**Table 1:** Thermophysical data of the four materials: Ar, CO, CO$_2$, and water ice. $T_t$ and $n_t$: triple-point temperature and number density.

	$T_t$ (K)	$n_t$ (nm$^{-3}$)	$T_b$ (K)	$T_c$ (K)	$n_c$ (nm$^{-3}$)	$T_c/T_t$
Ar	83.81	21.36	87.30	150.7	8.07	1.80
CO	68.13	18.27	81.66	132.9	6.53	1.95
CO$_2$	216.58	16.08	194.68	304.1	6.38	1.40
H$_2$O	273.16	33.42	373.12	647.1	10.76	2.37

$T_b$ boiling temperature at 0.1 MPa. $T_c$ and $n_c$: critical temperature and number density. Data taken from Refs.^61,62^.

In order to be able to compare these materials, we introduce units that are based on the molecular mass, *m*, and the triple-point temperature $T_t$ and number density $n_t$: We use a length scale of $\sigma =1/n_t^{1/3}$ and an energy scale $\epsilon$ given by $k_B T_t = 0.66\epsilon$, where $k_B$ is Boltzmann’s constant. The numerical prefactors are chosen such that the triple-point density and temperature of amorphous Ar are correctly reproduced if Ar is modeled by the well-known Lennard-Jones (LJ) potential

1 $$\begin{aligned} V(r) = 4 \epsilon \left[ \left( \frac{\sigma }{r} \right) ^{12} - \left( \frac{\sigma }{r} \right) ^{6} \right] . \end{aligned}$$

We use these triple-point data, since the NPs are below or around the triple point even if collision-induced heating is taken into account. The density for the liquid phase is used, since data seem to be more easily available for this phase and also because the structure of our amorphous NPs more closely resembles that of the liquid state. Note that the density of amorphous LJ material is $n= 1.00/\sigma ^3$^37^; for crystalline LJ solids, it is somewhat higher, $n= 1.086/\sigma ^3$^38^. The triple-point and critical temperature of LJ material are $0.66\epsilon$ and $1.32\epsilon$, respectively^39–41^; the latter value depends strongly on the cut-off radius of the potential^42^. For the convenience of the reader, we note that for the amorphous LJ solids used here, the specific surface energy amounts to $1.63 \epsilon /\sigma ^2$; the Young’s modulus is $46.4 \epsilon /\sigma ^3$ and the Poisson ratio 0.37^37^.

The values of $\epsilon$ and $\sigma$ thus determined are assembled in Table 2. Based on the molecular mass and these values, units for velocity, time and pressure are also provided. The differences in the ratio of critical to triple-point temperature can be taken as an indication of the differences in thermophysical properties between these ices. We emphasize that we do not recommend to use a LJ potential to describe CO, CO$_2$ or water. Rather we use these units to be able to compare the simulation results for the various materials.

**Table 2:** LJ parameters, $\epsilon$ and $\sigma$, and mass, *m*, of the four materials: Ar, CO, CO$_2$, and water. LJ units for velocity, ${\bar{v}}=\sqrt{\epsilon /m}$, time ${\bar{t}}=\sigma \sqrt{m/\epsilon }$, temperature, $T=\epsilon /k_B$, and pressure, ${\bar{p}}=\epsilon /\sigma ^3$, where $k_B$ is Boltzmann’s constant.

	$\epsilon$ (meV)	$\sigma$ (Å)	*m* (amu)	${\bar{v}}$ (m/s)	${\bar{t}}$ (ps)	${\bar{T}}$ (K)	${\bar{p}}$ (MPa)
Ar	10.94	3.60	40.0	162.4	2.219	127.0	37.4
CO	8.90	3.80	28.0	175.1	2.168	103.2	26.0
CO$_2$	28.28	3.97	44.0	249.0	1.591	328.2	72.8
H$_2$O	35.74	3.10	18.0	437.7	0.708	413.9	191.5

Ar NPs are used as a reference case; Ar atoms interact with a LJ potential, Eq. (1). For CO, we employ a potential optimized for the thermodynamic properties^43^; it is based on a two-center LJ potential augmented by dipole and quadrupole potentials. A similar potential is used for CO$_2$^44^, in which the dipole term is of course missing. These potentials are part of the open access database MolMod^45^; it treats the molecules as rigid, since at the low temperatures encountered, excitation of molecular vibrations is excluded. For water, we use the so-called monatomic water potential designed by Molinero and Moore^46^ since it gives a good representation of amorphous water ice^47,48^ and also the thermodynamic properties of other water phases^49^.

For all these potentials, amorphous solids are prepared by quenching from the melt^50,51^ to a final temperature of 50 K. Spherical NPs with radius $R=20$ nm are cut out of these amorphous solids and relaxed for 50 ps in an NVE ensemble. The Ar NP contains 818409 atoms, and the CO, CO$_2$ and water NPs 637392, 674288 and 1119528 molecules, respectively.

For the collision simulation, the amorphous NP is duplicated and put outside of the cut-off radius of the interaction of the original NP. Only central collisions were considered. We found that for all velocities in the range of 150–100,000 m/s, collisions between CO$_2$ NPs were always sticking; this finding is in agreement with earlier simulations on amorphous LJ NPs^52^ and our previous results on water-ice NPs^28^. We therefore present here only the results of a central collision with a relative velocity of $v= 1.70$ LJ units; this corresponds to velocities of 276 (298, 424, 742) m/s for Ar (CO, CO$_2$, H$_2$O) according to Table 2. This case is representative of a velocity which induces considerable NP deformation. Towards smaller velocities, all collision-induced effects—such as collision-induced heating and deformation—become less relevant. We note that collision velocities of nanoparticles in protoplanetary disks will on average be much smaller, in the range of 1 m/s or below^2^, and this applies also to most laboratory experiments^20^. At these small velocities, central collisions between NPs will be sticking, since the kinetic energy available after the collision does not allow the collision partners to overcome the attractive interactions and to separate from each other.

The molecular dynamics simulations are performed with the LAMMPS code^53^. Atomistic snapshots are generated with OVITO^54^.

## Results

### Effect of initial temperature

We study the effect of the initial temperature $T_0$ for water and Ar NPs only, since the simulation times of the CO and CO$_2$ NPs are by far more excessive—by around two orders of magnitude in computation time—due to the inclusion of electrostatic forces. We compare water NP collisions at $T_0=50$, 100, 150 and 200 K. For Ar we use $T_0=15$, 31, 46 and 61 K, since these have the same fraction of the triple-point temperature. NP size and collision velocity were always identical, as above.

**Figure 1:**
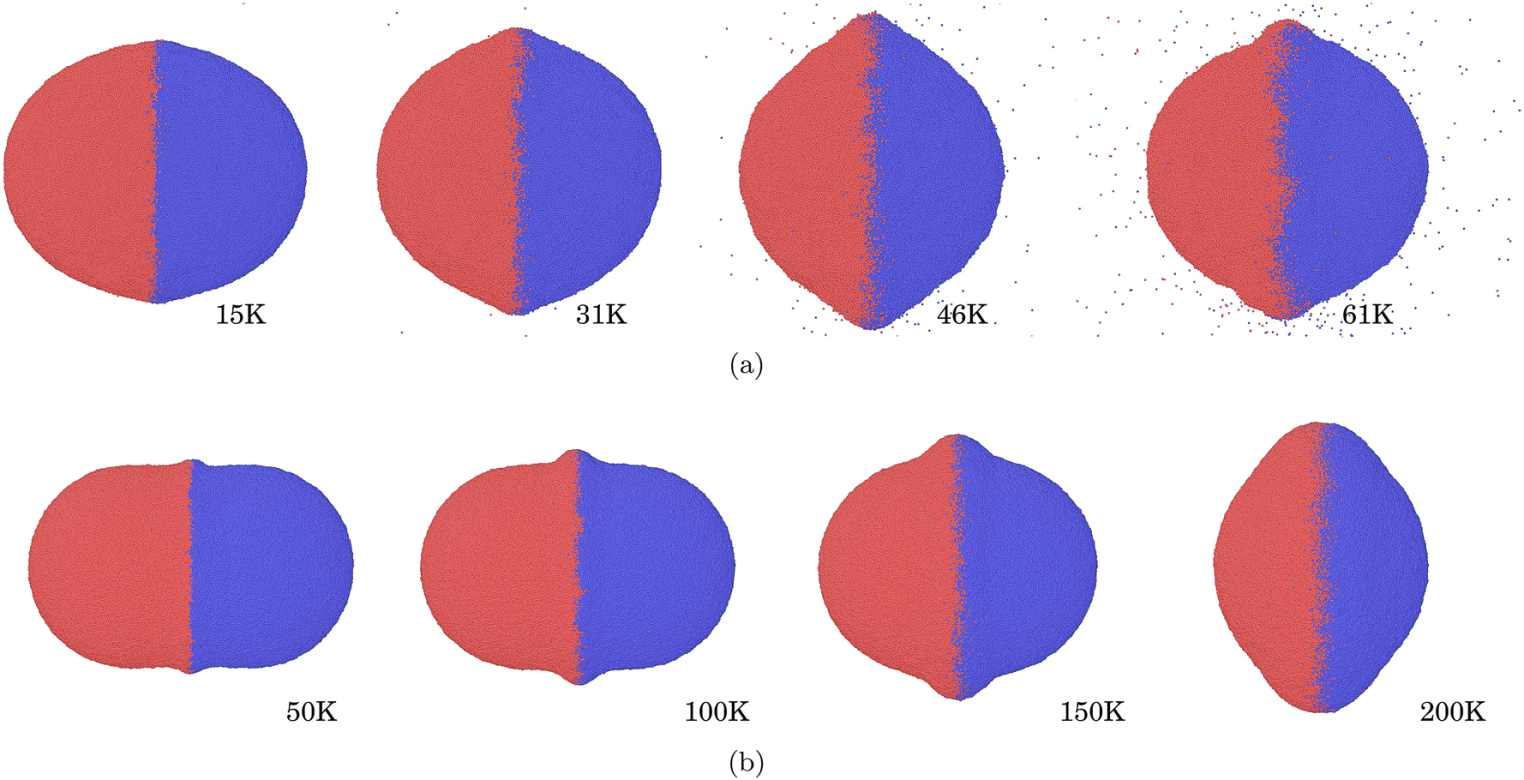
Configuration of the merged cluster for (**a**) Ar and (**b**) water NP collisions at the end of the simulation for the initial temperatures $T_0$ indicated. Color denotes initial NP affiliation. The snapshots in each row are at the same scale.

Figure 1 displays the final configurations of the merged cluster at a time of 324 ps. A strong effect of the initial temperature on the final shape is visible for both materials. With increasing $T_0$, the collision-induced deformation becomes stronger. While at the lowest temperature, the merged cluster resembles a stack of two spherical caps, a rim at the collision zone evolves and becomes larger with increasing $T_0$. At the higher temperatures, the material of the two NPs becomes mixed in the collision zone. While at low temperatures, the rim is formed in the collision zone, at the highest temperature, the rim for water has a rounded shape indicating strong material relaxation processes occurring in the aftermath of the collision. We note that the shape of the merged water cluster for the highest initial temperature, $T_0=200$ K, still evolves by decreasing its cross section in order to reduce its surface.

For Ar, a large number of atoms is ejected during the collision for the higher temperatures; this is in line with the larger sublimation rate of Ar as compared to H$_2$O. A detailed analysis shows that the number of ejecta increases exponentially with $T_0$, from 2 at 15 K to 66913 at 61 K, see the Supplementary Material (SM). Water NP collisions did not lead to any molecule ejection, not even at the highest temperature considered.

**Figure 2:**
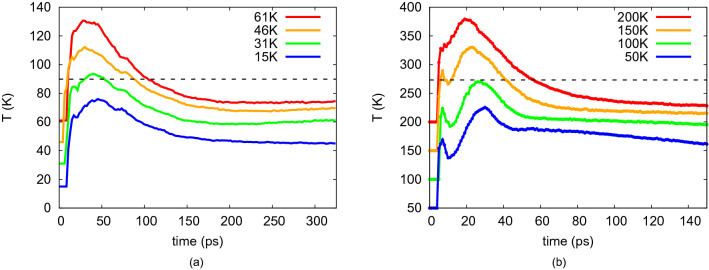
Time evolution of the temperature in the collision zone for (**a**) Ar and (**b**) water NP collisions for the initial temperatures $T_0$ indicated. The horizontal dashed line marks the triple-point temperature.

The material deformation under the collision is connected to the collision-induced temperature increase in the collision zone. We plot the temperature in the collision zone as a function of time in Fig. 2. To this end, we define the *collision zone* as a slab of width 1 nm at the center of mass of the collision system. The temperature of this slab—corresponding to the kinetic energy of all atoms in the collision zone—is then shown in Fig. 2. Initially, before any atoms are in the collision zone, the temperature is undefined. A strong increase in temperature during the collision is observed in all cases. For water, the temperature peak is split into two peaks, which, however, merge for the Ar system. The high temperature during the collision relaxes at later times, by heat conduction to the rear parts of the collided clusters and—in the case of Ar—by evaporation.

With the exception of the smallest $T_0$, the temperature reaches and even surpasses the triple-point temperature indicating local melting in the collision zone. This local heating and even melting explains the strong deformations of the merged cluster occurring in the collision zone. The maximum heating, $T(t) - T_0$, occurring under the collision is approximately independent of the initial temperature $T_0$ and amounts to 180 K for water and 60 K for Ar, see the SM. The final temperature in the collision zone, however, shows a strong dependence on $T_0$ in that it decreases monotonically with $T_0$. One reason hereto is that the increase in contact area with $T_0$ leads to a more efficient energy transfer out of the collision zone. More important, however, is the fact that the phase transformation occurring when the temperature surpasses the triple-point temperature costs latent heat and thus cools the collision zone. It should be noted that the strong collision-induced temperature increase occurs in the collision zone, where the two NPs interact; therefore at low initial temperature, the strongest deformation occurs only in the collision zone—such as the formation of a collision bulge in Fig. 1. At higher initial temperatures, the ice material becomes softer and the bulk of the NPs also undergoes deformation.

Further quantitative information on the processes occurring under the collision are provided in the SM where the evolution of the contact area and of the pressure in the collision zone are displayed. The pressure shows little variation with $T_0$, while the contact area increases with $T_0$ as discussed in connection with Fig. 1 above.

### Ice species

In this section, we study the collision of various ice NPs of the same temperature, $T_0=50$ K, and size, $R=20$ nm, impacting with the same scaled velocity.

**Figure 3:**
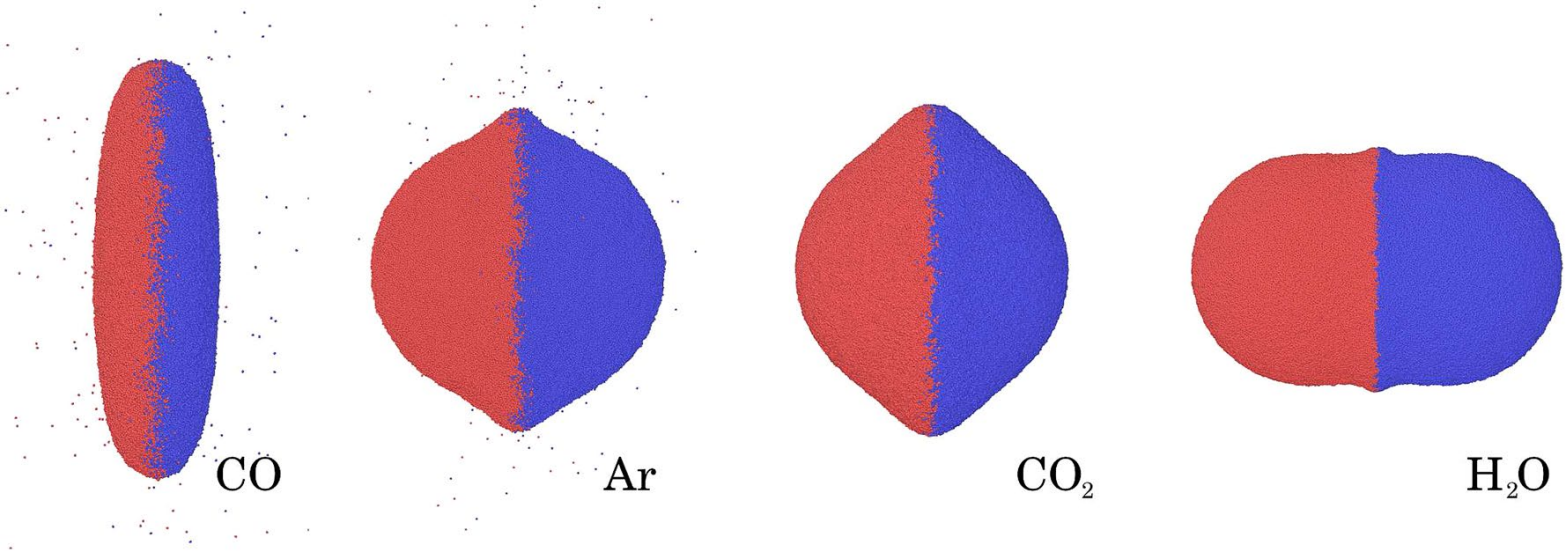
Configuration of the merged cluster at the end of the simulation for the species indicated. Color denotes initial NP affiliation. The snapshots are at the same scale.

**Figure 4:**
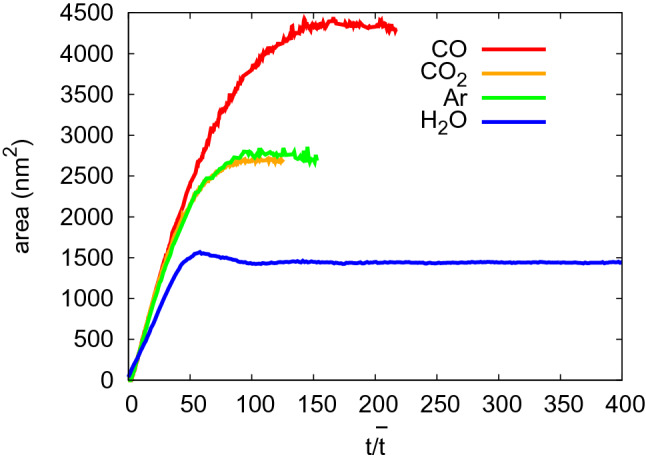
Evolution of the contact area for NP collisions with the species indicated with scaled time, $t/\bar{t}$.

Figure 3 visualizes the shape of the merged clusters at the end of the simulation. The snapshots were taken at the end of the simulation, when the contact area did not change any more, cf. Fig. 4 below. The CO NP shows the strongest deformation, in which the merged NP is of a pancake-like shape. This is in agreement with the small triple-point temperature of this material. CO$_2$ and Ar show similar deformations, even though their triple-point temperatures differ by a factor of 2.6. The rounded shape of the CO$_2$ rim gives evidence of post-collision relaxation in the material. With the highest triple-point temperature, H$_2$O exhibits the most undisturbed shape.

The Ar and CO NP collisions generate to ejecta, 530 atoms and 306 molecules for Ar and CO, respectively. Most of the ejecta are single atoms or single molecules. There are only 3 dimers for Ar and 2 for CO. Water and CO$_2$ do not produce any ejecta. Thus, the generation of ejecta follows the trend in triple-point temperatures, see Table 1, as it is plausible.

Figure 4 shows the time evolution of the contact area. If scaled times are used, the initial growth rate of the contact areas coincide; this demonstrates that the use of scaled variables allows to cover the processes during and immediately after the collision faithfully. The evolution at later times, however, shows strong deviations amongst the four systems that are in agreement with the qualitative discussion of the snapshots, Fig. 3, above. In particular, the water-ice NP is the stiffest and features a rebound phenomenon, i.e. the contact area shows a temporary maximum as the contact rim expands outward but then relaxes again to a smaller contact area. While CO and Ar show a similar final area, CO shows the largest growth. These results demonstrate that it is not only the thermophysical properties, $T_t$ and $n_t$ contained in Table 1 that predict the outcome of the collision, but also further elastic and plastic properties of the ices, and thus their equation of state.

**Figure 5:**
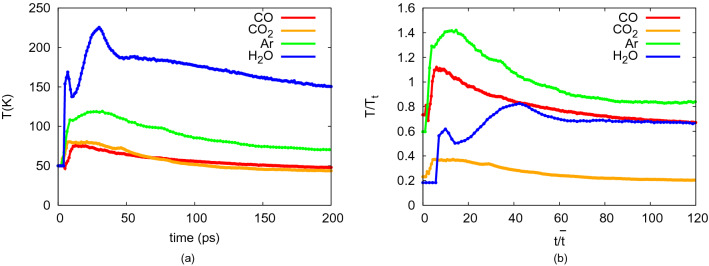
(**a**) Time evolution of the temperature in the collision zone for NP collisions with the species indicated. (**b**) displays the same data relative to the triple-point temperature, $T_t$.

Figure 5a shows the time evolution of the temperature in the collision zone; for convenience, Fig. 5b provides the same data in reduced units. In contrast to the contact areas, the time evolution of the temperatures differ strongly between the ice species. Ar features a stronger heating than CO, both in absolute terms and relative to the triple-point temperature. Note that CO is a molecular species whose rotational degrees fo freedom can be excited; the correspondingly larger heat capacity explains the relatively modest heating of CO. Therefore, also the absolute temperature increase in CO$_2$ is comparable to that of CO. Water features the highest temperature increase in absolute terms. For the monatomic water model used here, rotational excitation is not included, thus artificially reducing the degrees of freedom available.

Note that intramolecular vibrational excitation is not included in any of the molecular models used since these were considered rigid. This assumption appears justified at the small temperatures encountered after the collision. All intermolecular vibrations are of course fully taken into account in the simulation.

**Figure 6:**
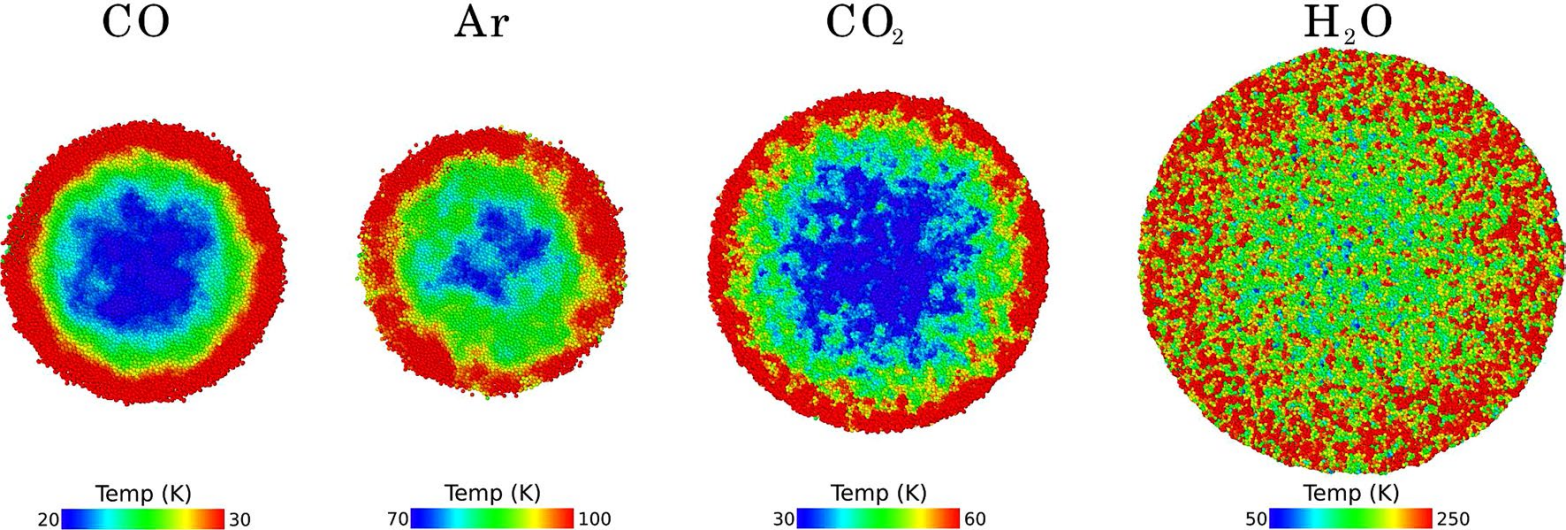
Temperature profile in the collision zone for NP collisions with the species indicated. Data taken at a time of 30 ps. The color scale changes for each species due to the different heating. The snapshots are at the same scale.

Only water features a double peak in the temperature evolution. The first peak at around 10 ps coincides with the maximum of the pressure in the collision zone, see the SM, and is therefore immediately induced by the collision. The second peak, at around 30 ps, shows up when the pressure is already decreasing. It is caused by heating at the expanding rim. We display the temperature distributions in the collision zone at the time of 30 ps in Fig. 6. For all species, the heating is maximum in the expanding rim, even if the size of the effect strongly differs with the species, ranging from around 10 K for CO to more than 100 K for water. At later times, the temperature distribution in the collision zone homogenizes. This figure thus demonstrates that heating in the expanding rim of the collision zone may contribute strongly to heating.

Heating is caused by friction during the radial expansion flow. As radial velocities are largest close to the rim, heating is largest there as well. The kinetic energy in the radial expansion flow is comparable to the temperature increase in the collision zone. For the example of water, the average expansion velocity amounts to around 360 m/s for water, see Fig. 4, which corresponds to 12 meV per molecule; in comparison, the temperature increase of 100 K corresponds to 8.6 meV per molecule.

**Figure 7:**
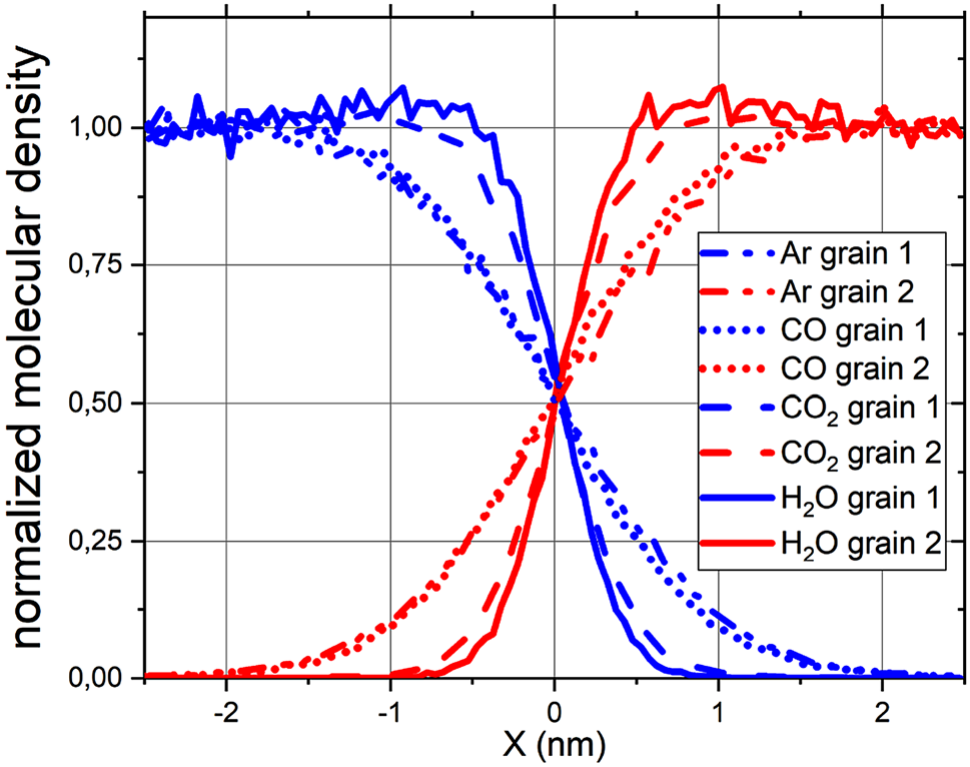
Mixing profiles of the NPs after collision. *X* measures the distance from the center of mass of the collided clusters, i.e., the center of the collision zone. The ordinate shows the number density of molecules originally affiliated to grain 1 and grain 2, normalized to their original bulk values. The colors denote initial NP affiliation as in Fig. 3.

We studied the amount of material intermixing between the two colliding NPs in the collision zone, see Fig. 7 by plotting the number density of molecules originally affiliated to grain 1 and grain 2 as a function of the distance to the center of the collision zone. Molecules are displaced between the grains by the collision, generating a mixed interface region. The water NPs even show a slight compression in the collision zone. Mixing is stronger in Ar and CO, where the mixed region has a width of 2 nm and includes 50,000 molecules (for CO), while it is less pronounced in H$_2$O and CO$_2$ with a width of 1 nm and 11,500 molecules (for water). Thus, the mixing efficiency correlates inversely with the triple-point temperatures of the materials; this appears plausible if mixing is caused by diffusional motion in the collision-heated contact zone. Note that Fig. 6 shows that only Ar and CO reach temperatures higher than the triple-point temperature after the collision.

These numbers demonstrate that collision-induced mixing may be a strong effect even in cases of minor NP deformation. Such mixing processes may be relevant for instance for NPs whose surfaces are contaminated by adsorbates; these would mix and might react in the collision zone redistributing the adsorbates to the interior of merged clusters and providing new molecular species as reaction products.

## Summary

We studied the collision behavior of amorphous cryogenic ice NPs and found the following features.

1. Nanoparticles of the size considered here—a few ten nm—are always sticking under collisions since energy dissipation in the collision is strong.
2. For higher collision velocities, they are strongly deformed under the collision. The deformation is plastic.
3. The temperature at which the collision occurs strongly influences the collision outcome. Partial melting of the merged cluster in the collision zone contributes to energy dissipation and deformation.
4. The high plasticity is caused by the high elastic and plastic softness of these cryogenic ices.
5. The number of ejecta emitted during the collision follows the trend in triple-point temperatures and increases exponentially with the NP temperature.
6. In the collision zone, material is mixed between the two colliding NPs. Mixing increases if the local temperature approaches or surpasses the triple-point temperature.
7. Even at comparable collision conditions, the collision outcomes of the ices investigated here—Ar, CO, CO$_2$ and H$_2$O—differ from each other due to differences in the thermophysical properties of these materials.

The amorphous 20-nm sized ice NPs did not show any bouncing, in contrast to experiments on larger (μm-sized) CO$_2$ and H$_2$O clusters^14,24^. We argue that there may be several reasons for this difference.

i. Our ice particles are clean, while in experiments, the surface of the colliding NPs may be contaminated. Such contaminations reduce the surface energy of the NPs; this effect has been discussed in great detail by Kimura et al.^55^ for the case of silica particles, where the effect of adsorbates on the surface energy may reach up to two orders of magnitude. A reduction of the NP surface energy will increase the tendency for bouncing.
ii. Also, real surfaces will be rough; in addition, the NP shape may deviate from a sphere. Both effects will influence the bouncing behavior.
iii. It has been shown that both surface adhesion and viscous dissipation processes lead to dissipation of kinetic energy, and experiments and simulations for micro-scale impacts show that the bouncing velocity threshold increases as the NP radius decreases^56^. This is because of the competition for a given NP radius, between adhesion, which increases restitution coefficient as velocity increases, and dissipation, which has the opposite effect^30^. We note that a similar argument for crystalline NPs was recently published^57,58^.
iv. Finally, our present study focused on central collisions, while in experiment and in nature large impact parameters will occur. Large impact parameters decrease the tendency for sticking as only parts of the colliding NPs come into close contact and experience their mutual attraction^52,59^.

Recently, it was shown that energy dissipation in granular aggregates of water ice leads to different aggregation and fragmentation outcomes^60^. The present work indicates that phase transformation should be considered as a possible energy dissipation channel also for aggregate collisions.

In future work, it will be interesting to extend the present results to larger NPs in order to see whether the differences in the bouncing behavior observed experimentally for microscopic grains start to evolve for NPs.

## Supplementary Information

Supplementary Information.

## Data Availability

All data used for this study are contained in this article.
