# Supplementary material for: Collisions between CO, CO2, H2O and Ar ice nanoparticles compared by molecular dynamics simulation
Source: Sci Rep. 2022 Aug 16;12:13858. doi: 10.1038/s41598-022-18039-5 (PMC9381553; doi:10.1038/s41598-022-18039-5)
Supplement: Supplementary file 1 — Supplementary Information. [file 41598_2022_18039_MOESM1_ESM.pdf]

Collisions between CO, CO_2, H_2O and Ar ice nanoparticles compared by molecular dynamics simulation

Maureen L. Nietiadi,[1] Yudi Rosandi,[2] Eduardo M. Bringa,[3,4] and Herbert M. Urbassek[1]

[1] *Physics Department and Research Center OPTIMAS, University Kaiserslautern, Erwin-Schrödinger-Straße, D-67663 Kaiserslautern, Germany*
[2] *Department of Geophysics, Universitas Padjadjaran, Jatinangor, Sumedang 45363, Indonesia*
[3] *CONICET and Facultad de Ingenería, Universidad de Mendoza, Mendoza 5500, Argentina*
[4] *Centro de Nanotecnología Aplicada, Facultad de Ciencias, Universidad Mayor, Santiago 8580745, Chile*
(Dated: July 25, 2022)

Effect of initial temperature

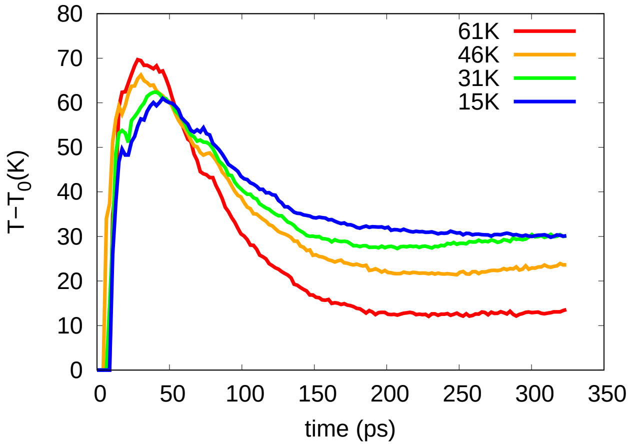

(a)

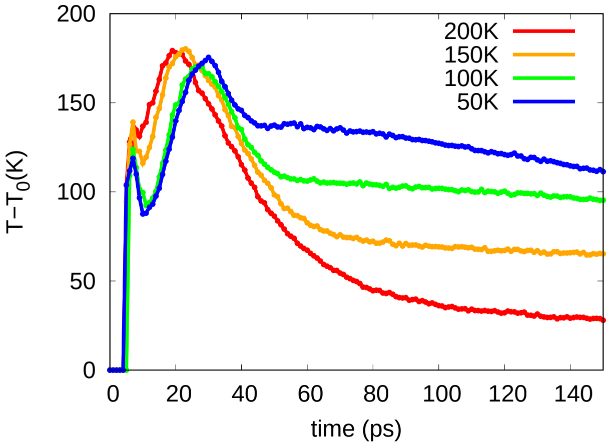

(b)

FIG. S1: Time evolution of the temperature, $T(t)$, in the collision zone, relative to the initial temperature T_0, for (a) Ar and (b) water cluster collisions for the initial temperatures T_0 indicated. The horizontal dashed line marks the triple-point temperature.

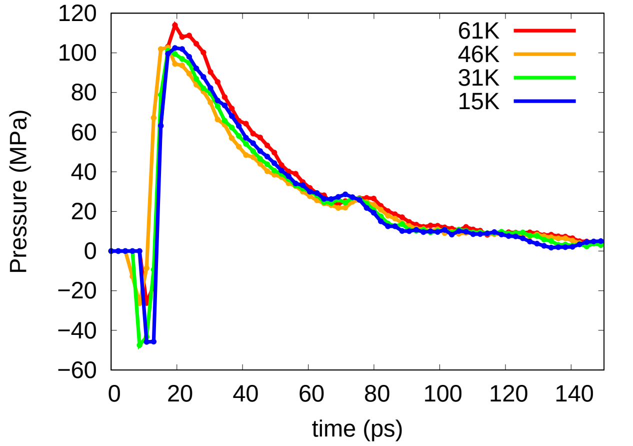

(a)

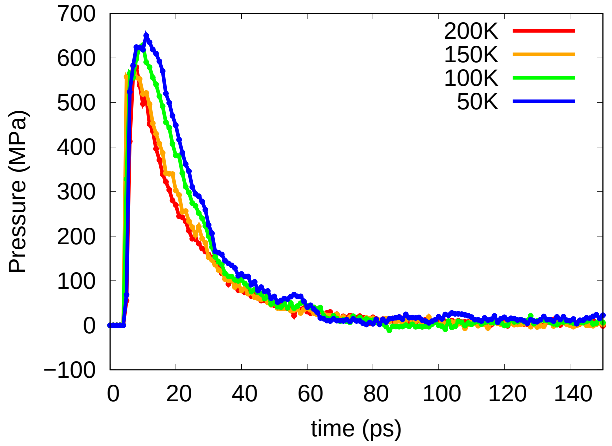

(b)

FIG. S2: Time evolution of the pressure in the collision zone for (a) Ar and (b) water cluster collisions for the initial temperatures T_0 indicated.

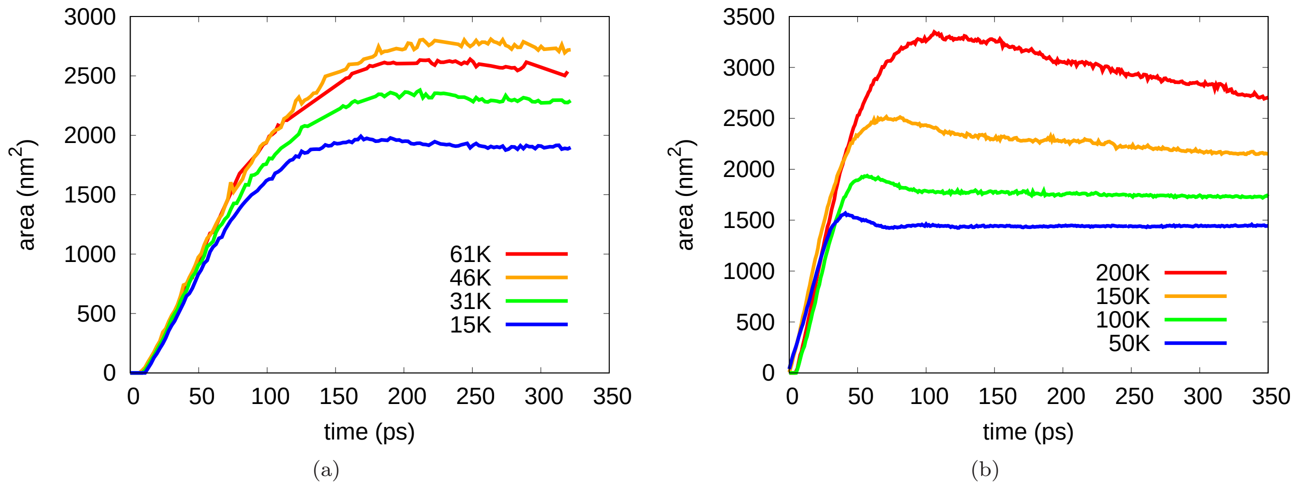

FIG. S3: Time evolution of the contact area in the collision zone for (a) Ar and (b) water cluster collisions for the initial temperatures T_0 indicated.

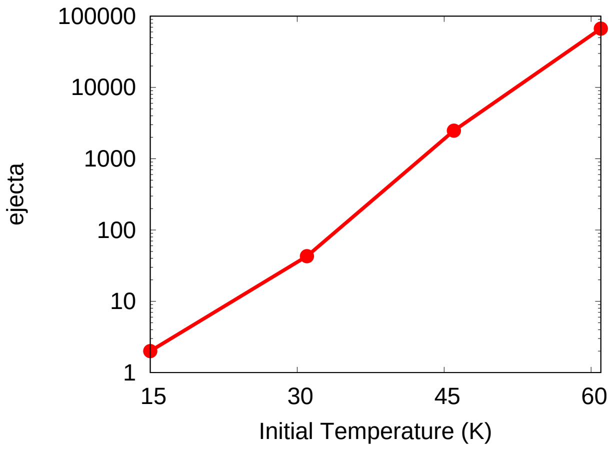

FIG. S4: Dependence of the number of ejecta for the Ar NP collision on the initial temperature T_0.

Ice species

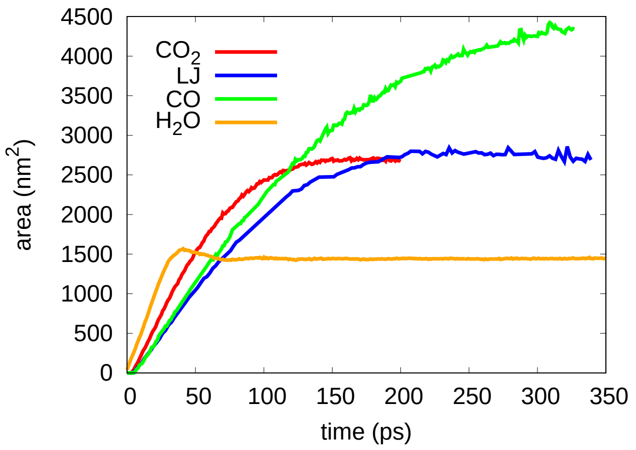

FIG. S5: Evolution of the contact area for NP collisions with the species indicated with time.
